# MiR-7 Promotes Epithelial Cell Transformation by Targeting the Tumor Suppressor KLF4

**DOI:** 10.1371/journal.pone.0103987

**Published:** 2014-09-02

**Authors:** Karla F. Meza-Sosa, Erick I. Pérez-García, Nohemí Camacho-Concha, Oswaldo López-Gutiérrez, Gustavo Pedraza-Alva, Leonor Pérez-Martínez

**Affiliations:** Laboratorio de Neuroinmunobiología, Departamento de Medicina Molecular y Bioprocesos, Instituto de Biotecnología, Universidad Nacional Autónoma de México, Cuernavaca, Morelos, México; CNRS UMR7275, France

## Abstract

MicroRNAs (miRNAs) are endogenous small non-coding RNAs that have a pivotal role in the post-transcriptional regulation of gene expression and their misregulation is common in different types of cancer. Although it has been shown that miR-7 plays an oncogenic role in different cellular contexts, the molecular mechanisms by which miR-7 promotes cell transformation are not well understood. Here we show that the transcription factor KLF4 is a direct target of miR-7 and present experimental evidence indicating that the regulation of KLF4 by miR-7 has functional implications in epithelial cell transformation. Stable overexpression of miR-7 into lung and skin epithelial cells enhanced cell proliferation, cell migration and tumor formation. Alteration of these cellular functions by miR-7 resulted from misregulation of KLF4 target genes involved in cell cycle control. miR-7-induced tumors showed decreased p21 and increased Cyclin D levels. Taken together, these findings indicate that miR-7 acts as an oncomiR in epithelial cells in part by directly regulating KLF4 expression. Thus, we conclude that miR-7 acts as an oncomiR in the epithelial cellular context, where through the negative regulation of KLF4-dependent signaling pathways, miR-7 promotes cellular transformation and tumor growth.

## Introduction

Krüppel-like factor 4 (KLF4) is a transcription factor (TF) expressed in the epithelium of a variety of tissues including the intestinal tract [1], skin [2], cornea [3] and lung [4]. At the sequence level, the *klf4* gene shares a 90% identity between human and mouse and it codes for a 55 KDa protein [5]. KLF4 has important roles in diverse biological processes such as cellular proliferation, differentiation, apoptosis, development and in tissue homeostasis maintenance [2], [6]–[11]. Importantly, KLF4 can either activate or repress the transcription of its target genes [10]–[12]. Thus, depending on the genetic and epigenetic context of the cell type, KLF4 can act as a tumor suppressor or as an oncogene [7], [10], [11]. This opposite functions are attributed to KLF4 capability of negatively regulate the transcription of the cell cycle progression regulator *cyclin D1* [13], [14] and positively regulate the transcription of cell cycle inhibitors such as *p21* [15], [16] and *p27* [15]. The activity of KLF4 as a tumor suppressor has been suggested in different types of cancers in which its expression is downregulated such as leukemia [17], [18], gastric [19], colorectal [20], esophageal [21], [22], bladder [23] and non-small-cell lung carcinomas [24]. Moreover, it has been reported that the absence of KLF4 promotes tumor development in mice treated with carcinogenic agents [25]. Accordingly, KLF4 protein levels are almost undetectable in biopsies obtained from patients with non-melanoma skin cancers such as squamous cell carcinoma (SCC) and basal cell carcinoma (BCC) [25]. In sharp contrast, KLF4 acts as an oncogene in a breast cancer context where elevated KLF4 expression has been observed [26]. Although it is clear that the control of KLF4 protein levels is crucial to prevent carcinogenesis, the molecular mechanisms involved in this process start to be elucidated.

miRNAs are small endogenous RNAs of ∼19–21 nucleotides (nt) capable of guide the post-transcriptional silencing of their target mRNAs through base pairing encompassing mature miRNA's 2–8 bases (seed sequence) and the mRNA 3′ UTR [27]. miRNA silencing of a target mRNA could be achieved either by target degradation or by translational inhibition [27]. miRNAs play a key role in a wide variety of cellular processes which require an exquisite spatio-temporal regulation of gene expression including development [28]–[30], metabolic processes [31], cellular differentiation [29], [32], [33], cellular proliferation and programmed cell death [31]. Therefore, it is not surprising that deregulation of miRNAs expression has been reported in different scenarios where cellular homeostasis is altered such as in cancer. Indeed, miRNAs also function as tumor suppressors or as oncogenic miRNAs (oncomiRs) [34]. miR-10b [35], miR-206 [36] and miR-103/107 [37] have been characterized as oncomiRs as their overexpression in esophageal and colorectal cancer correlates with enhanced proliferative and/or metastatic phenotypes that result from the downregulation of the tumor suppressor KLF4 [35]–[37]. In contrast, it has been recently shown that the loss of KLF4 negative regulation by the miR-7, in cancer stem-like cells (CSCs) derived from breast cancer, enhanced their metastatic capacity towards the brain [38]. Contrary to its tumors suppressor function in breast cancer, miR-7 has been previously reported to function as an oncomiR in other cellular contexts including epithelial lung carcinoma [39] and renal cell carcinoma (RCC) of epithelial cells [40]. The oncogenic role of miR-7 in epithelial lung carcinoma results in part, from silencing the Ets2 transcriptional repressor factor which controls cell proliferation via the Ras/ERK-mediated pathway [39]. Based on the tumor suppressor role of KLF4 in cancer of various epithelial tissues and the reported oncogenic activity for miR-7 in epithelial lung carcinoma and epithelial RCC; we hypothesized that during the transformation process of epithelial cells, the negative regulation of KLF4 by miR-7 results in a carcinogenic process. Here, we demonstrated the functional interaction for miR-7 with a predicted binding site within the KLF4 3′ UTR. Consistently with previous reports suggesting an oncogenic role for miR-7 in a lung epithelial cellular context [39], we show that miR-7 through targeting KLF4, induced survival, proliferation and migration of HaCaT and A549 cells. Moreover, miR-7 augmented the transformed phenotype of A549 cells and induced the formation of tumors in nude mice by altering the expression of the known KLF4 target genes, p21 and Cyclin D1. Thus, we conclude that miR-7 has an important role in the regulation of KLF4-dependent signaling pathways in the epithelial cellular context.

## Results

### The KLF4 3′ UTR contains two evolutionary conserved binding sites for miR-7

**Figure 1:**
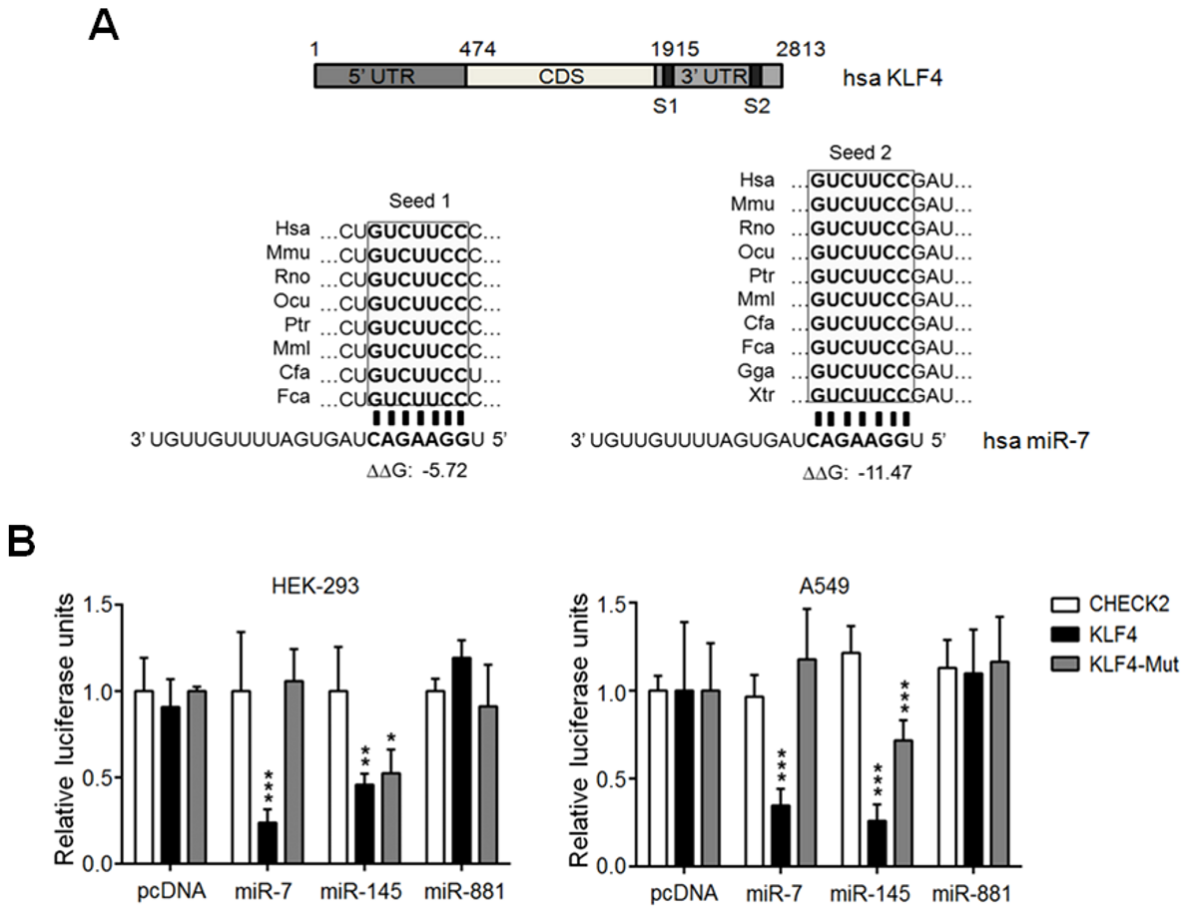
KLF4 3′ UTR contains two evolutionary conserved binding sites that mediate the interaction with miR-7. (A) Schematic representation of the human KLF4 mRNA indicating the relative positions of the two identified miR-7 binding sites denoted as Seed 1 (S1) and Seed 2 (S2) located respectively, at positions 66–72 nt and 574–580 nt relative to the beginning (1915 nt) of the human KLF4 3′ UTR. Both S1 and S2 are phylogenetically conserved as shown in sequence alignments of different organisms. S1 is conserved in mammals and presents a thermodynamic stability (ΔΔG) of −5.72 kcal/mol while; S2 is conserved in all vertebrates and possesses a ΔΔG of −11.47 kcal/mol. (B) HEK-293 and A549 cells were co-transfected with 200 ng of pc/miR7, pc/miR145, pc/miR881 or empty vector (pcDNA) together with 100 ng of the wt KLF4 3′ UTR (KLF4), the mutated version of KLF4 3′ UTR for the second miR-7 binding site (KLF4-Mut) or empty vector (CHECK2). Luciferase activity was determined 48 hours post-transfection and relative luciferase units were determined as described in materials and methods. Results represent the mean of at least six independent experiments. ***p<0.001, **p<0.01, *p<0.05 *vs.* pcDNA.

Previous studies have demonstrated that KLF4 expression can be regulated at the post-transcriptional level and that regulation of KLF4 protein levels is important for cell proliferation and differentiation [41]–[43]. Accordingly, deregulation of some miRNA:KLF4 circuits results in carcinogenic phenotypes [35]–[37]. Given that KLF4 protein levels are diminished in SCC and BCC [25], we asked whether KLF4 could be regulated post-transcriptionally by miRNAs during epithelial cell transformation. Using different bioinformatic tools [44]–[47], we identified several miRNAs with potential binding sites conserved between the 987 nt mouse (NM_010637) and the 899 bp human KLF4 3′ UTR (NM_004235) and high thermodynamic score (Table S1). Among the selected miRNAs, miR-7 was ranked as the best candidate with two binding sites with perfect complementarity in the seed region at two different positions within the 3′ UTR of the human and the mouse KLF4 mRNAs (Figure 1A). These two miR-7 binding sites previously described by Okuda *et al.* [38] are phylogenetically conserved among different organisms (Figure 1A).

### KLF4 3′ UTR is directly targeted by miR-7

To validate our bioinformatic analyses, we cloned 975 nt of the mouse wt KLF4 3′ UTR (psi/KLF4) containing the two putative miR-7 binding sites downstream of the *Renilla* luciferase reporter gene. As the mouse pre-miR-7a (mmu-miR-7a) and the human pre-miR-7 (hsa-miR-7) give rise to the same mature miR-7, the mouse pre-miR-7a was cloned into the pcDNA expression vector under the control of the cytomegalovirus (CMV) promoter (pc/miR7). HEK-293 and A549 cells were transfected and luciferase activity was evaluated. Despite the fact that both cell lines expressed endogenous miR-7 (Figure S1A), miR-7 overexpression (Figure S1B) decreased luciferase activity derived from the wt KLF4 3′ UTR vector in both HEK-293 and A549 cells to a similar extent (Figure 1B). The reduction in luciferase activity observed in miR-7 expressing cells was similar to that resulting from miR-145 expression, a *bona fide* KLF4 negative regulator [43]; while miR-881 expression, which contains no binding sites on the KLF4 3′ UTR did not affect luciferase activity (Figure 1B, left and right panel). Given that the second binding site for miR-7 within the KLF4 3′ UTR was thermodynamically stable to interact with its target sequence (ΔΔG of −11.47) and that is highly conserved in vertebrates, we evaluated the specificity of the miR-7:KLF4 3′ UTR interaction. For this, the seed sequence of the second miR-7 binding site (GTCCTTCC) was mutated (CT for AA). As expected, this mutation prevented the miR-7 negative effect on luciferase activity in both cellular contexts (Figure 1B). In contrast, miR-145 expression resulted in decreased luciferase activity derived from the mutant KLF4 3′ UTR vector (Figure 1B), indicating that the Seed 2 is necessary for the miR-7 mediated KLF4 repression and that the mutation on Seed 2 did not interfere with other miRNAs mediating KLF4 repression.

### Downregulation of KLF4 protein levels by miR-7

miRNAs are known to repress expression of their target genes either by mRNA degradation or by translational inhibition [27]. Accordingly, in contrast to empty vector (pcDNA) or miR-881 transfected cells, the protein levels of KLF4 decreased in a dose-dependent manner in HEK-293 cells overexpressing miR-7 or as expected, in cells overexpressing miR-145 (Figure S2). However, the maximum silencing capacity was specific for each miRNA. While 1 µg of miR-7 was necessary to produce a 64% repression of KLF4 protein levels, 200 ng of miR-145 were enough to get a similar repressive effect (62%). Interestingly, 50 ng of miR-145 showed a more repressive effect over KLF4 protein levels than 100 or 200 ng (Figure S2). Given that miRNAs also positively-regulate gene expression by targeting promoter elements [48], this apparent contradictory data could be due to a positive effect on KLF4 gene expression mediated by high miR-145 concentrations specifically, since this effect was not observed with any other overexpressed miRNA.

As for HEK-293 cells, miR-7 overexpression in HaCaT and A549 cells mediated downregulation of KLF4 protein levels (Figure S3B and S4B). These results indicate that miR-7 negatively regulates endogenous KLF4 protein levels independently of the cellular context and are in agreement with those published by Okuda and colleagues while our manuscript was in preparation showing that miR-7 targets KLF4 in breast CSCs [38].

### miR-7 enhances proliferative potential of HaCaT and A549 cells

**Figure 2:**
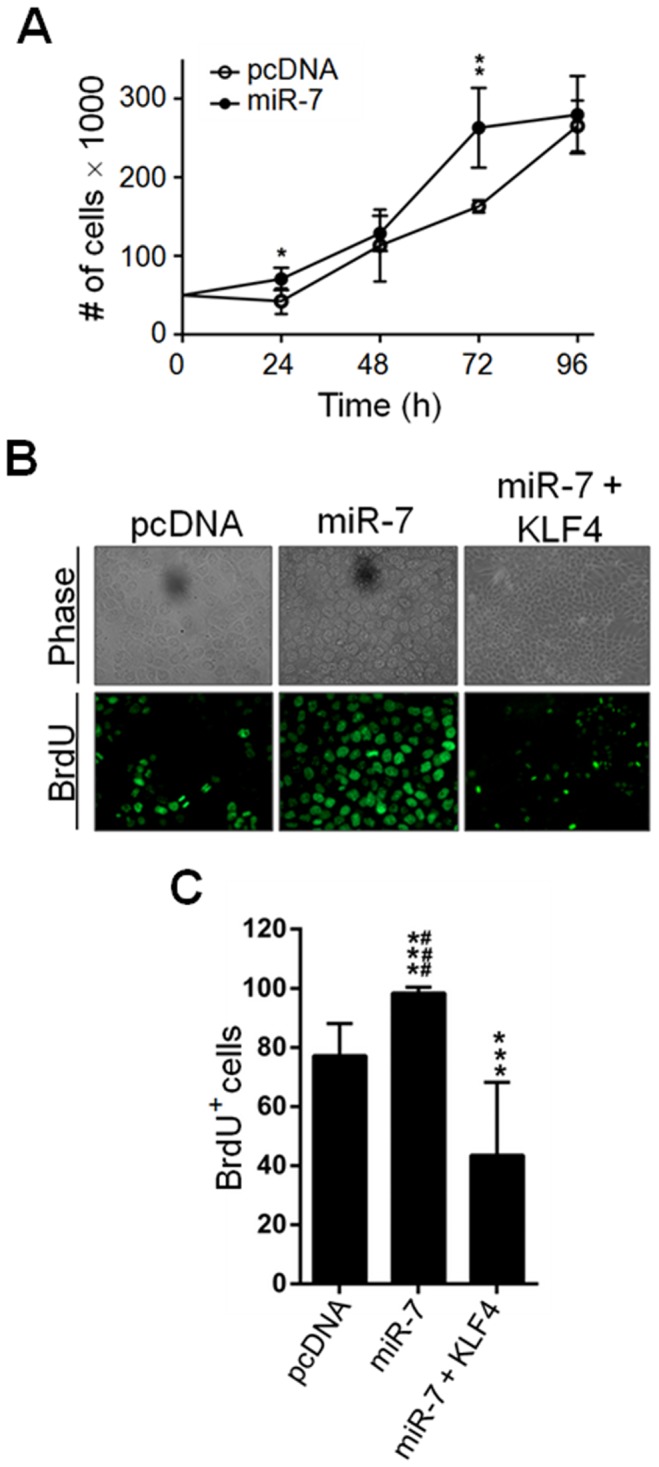
miR-7 overexpression promotes HaCaT cell proliferation and entry to the S phase of the cell cycle. (A) 5×10^3^ cells of independent miR-7 or pcDNA overexpressing clones were plated onto 24 well culture plates and counted every 24 hours during four consecutive days. The percentage of cells entering to S phase of the cell cycle was determined by BrdU incorporation as described in materials and methods. BrdU+ cells were visualized under UV microscopy (B), cells were counted and the results plotted (C). Results represent the mean of three independent experiments performed with five or six pcDNA, miR-7 or miR-7+KLF4 overexpressing independent clones. ***p<0.001 and **p<0.01 *vs.* pcDNA, ###*p*<0.001 *vs.* miR-7+KLF4.

Given its role as a tumor suppressor, KLF4 protein levels are decreased in tumors derived from epithelial cells, however the molecular mechanisms involved in KLF4 downregulation in oncogenic epithelial cells, has not been explored. In contrast, miR-7 has been described as an oncomiR in epithelial RCC [40] and in epithelial lung cancer cells in part by targeting the Ets2 TF [39]. Consequently, we asked whether miR-7 could play an oncogenic role by negatively regulating KLF4 expression during epithelial cell transformation. Thus, we generated stable clones of the non-differentiated human keratinocytes HaCaT cell line overexpressing miR-7 (Figure S3) and evaluated their proliferative capacity. There was no difference in the proliferation rate between miR-7 and pcDNA transfected clones at 24 or 48 hours of culture (Figure 2A); however, after 72 hours a significant increase in the cell number of miR-7 overexpressing clones compared to pcDNA transfected clones was observed (Figure 2A). Given that the miR-7 expressing clones reached confluence at 72 hours after plating while the pcDNA transfected clones did it after 96 hours in culture, the difference in cell numbers was lost after 96 hours in culture (Figure 2A). To corroborate that miR-7 expression promotes cell cycle progression, the cell cycle profile of stable miR-7 expressing HaCaT cells was assessed by flow cytometry. pcDNA and miR-7 expressing cells showed similar cell cycle profiles after growth factors deprivation (Table 1 and Figure S5). However, 12 hours after growth factors addition, a lower percentage of miR-7 expressing cells was observed at the G1 phase compared to pcDNA transfected cells (miR-7 24.53±5.51% *vs.* pcDNA 34.1±7.61%; Table 1 and Figure S5) and a significant increase in the percentage of cells at the G2/M phase was observed in the miR-7 expressing cells compared to pcDNA transfected cells (miR-7 20.83±1.85% *vs.* pcDNA 13.8±1.85%; p<0.01; Table 1 and Figure S5). At 24 hours post-arrest, the number of miR-7 expressing cells at the G2/M phase of the cell cycle was also higher than that observed for the pcDNA transfected cells (miR-7 17.27±1.11% *vs.* pcDNA 10.99±2.52%; p<0.05; Table 1 and Figure S5). These results indicate that miR-7 expression enhanced HaCaT proliferative capacity. To confirm that miR-7 promoted cell cycle entry, cells entering into S-phase were quantified by BrdU incorporation. Almost 100% of the miR-7 expressing cells were BrdU positive (98.33±2%), while only around 70% of the pcDNA transfected cells incorporated BrdU (77.18±10.85%) (Figure 2B and 2C). To confirm that the effect of miR-7 on cell proliferation is KLF4-dependent, we co-expressed miR-7 and KLF4 (Figure S3). miR-7+KLF4 co-expressing cells showed a lower percentage of BrdU positive cells (43.54±24.69%) to that of pcDNA transfected cells (77.18±10.85%). Thus, these results indicate that KLF4 expression reversed miR-7 effect on cell cycle progression (Figure 2B and 2C).

**Table 1:** miR-7 overexpression promotes cell cycle progression.

	G1	S	G2/M
pcDNA 0 h	38.37±1.91%	52±2.18%	14.53±1.50%
miR-7 0 h	33.13±1.8%*	51.33±2.43%	14.85±2.42%
pcDNA 12 h	34.1±7.61%	52.17±9.12%	13.8±1.85%
miR-7 12 h	24.53±5.51%	55.27±3.52%	20.83±1.85%**
pcDNA 24 h	39.3±11.23%	48.7±11.46%	10.99±2.52%
miR-7 24 h	30.06±1.60%	53.97±0.60%	17.27±1.11%*

Values are means ± s.d.; h, hours. n = 3,

**p<0.01,

*p<0.05 *vs.* pcDNA values.

**Figure 3:**
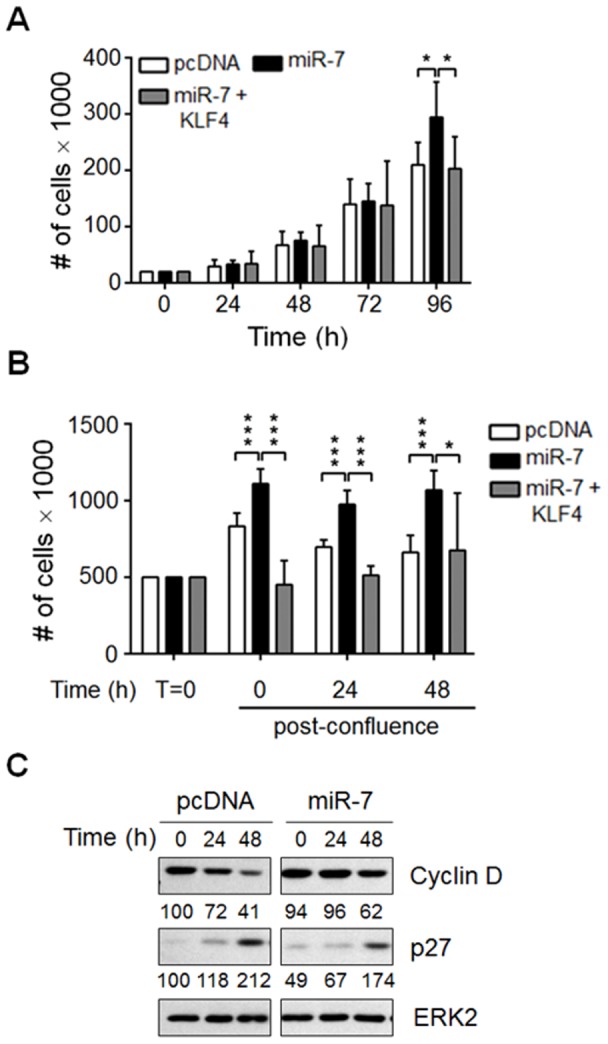
miR-7 overexpression promotes A549 cell proliferation through negatively regulating KLF4. (A) 2×10^4^ cells of pcDNA, miR-7 and miR-7+KLF4 overexpressing clones were plated in 24 well culture plates. Cells were counted every 24 hours during four consecutive days. (B) Once A549 cells reached confluence (T = 0), cell numbers were determined each 24 hours during two consecutive days. Fresh medium was added 24 hours post-confluence. Results represent the mean of six independent experiments performed with five or six pcDNA, miR-7 or miR-7+KLF4 overexpressing independent clones. ***p<0.001, *p<0.05 *vs.* pcDNA. (C) Protein levels of KLF4 target genes products. pcDNA and miR-7 expressing A549 cells were harvested at 0, 24 and 48 hours post-arrest, total cell extracts were prepared and Cyclin D and p27 protein levels were determined by Western blot analysis. ERK2 protein was used as loading control. Numbers denote the protein levels as the percentage of each condition relative to the pcDNA cells at 0 hours post-arrest. A representative Western blot of three independent experiments is shown.

We also tested whether miR-7 could induce the proliferative capacity of another epithelial cell line. pcDNA and miR-7 expressing clones from the human alveolar adenocarcinoma A549 cell line (Figure S4) showed a similar proliferation rate even after 72 hours in culture (Figure 3A). Nonetheless, 96 hours after plating, the miR-7 expressing clones showed significantly higher cell numbers than the pcDNA transfected clones (Figure 3A). Again, co-expression of KLF4 and miR-7 in A549 cells reduced the proliferation rate to levels similar to those observed in pcDNA transfected cells (Figure 3A) indicating that miR-7 promotes cell proliferation by targeting KLF4 in HaCaT and A549 cells and that miR-7 might function as an oncomiR in epithelial cells [39], [40].

Cells undergoing transformation are able to grow despite growth factors deprivation and space-limiting conditions. The capacity of transformed mouse fibroblasts to proliferate in serum withdrawal conditions correlates with Cyclin D expression [48]. To test whether miR-7 expression promotes proliferation in space- and nutrient-limiting conditions, A549 cells were allowed to reach confluence. According with the data presented above, although the three cell types reached confluence at the same time (T = 0), miR-7 expressing cells showed a significant increase in the cell number compared to pcDNA and miR-7+KLF4 transfected cells at all time points assayed (Figure 3B). This can be explained by the fact that, in contrast to pcDNA and miR-7+KLF4 cells, miR-7 expressing cells were able to grow on top of each other forming foci (data not shown). pcDNA and miR-7 expressing clones did not further increase in cell number after 24 hours post-confluence if not that decreased (Figure 3B). Interestingly, the addition of fresh medium at 24 hours post-confluence prevented the decline in cell number of miR-7 expressing clones but not that of pcDNA and miR-7+KLF4 clones (Figure 3B), suggesting that in limiting space conditions, miR-7 promotes cell proliferation and that this effect is reversed by KLF4 expression.

**Figure 4:**
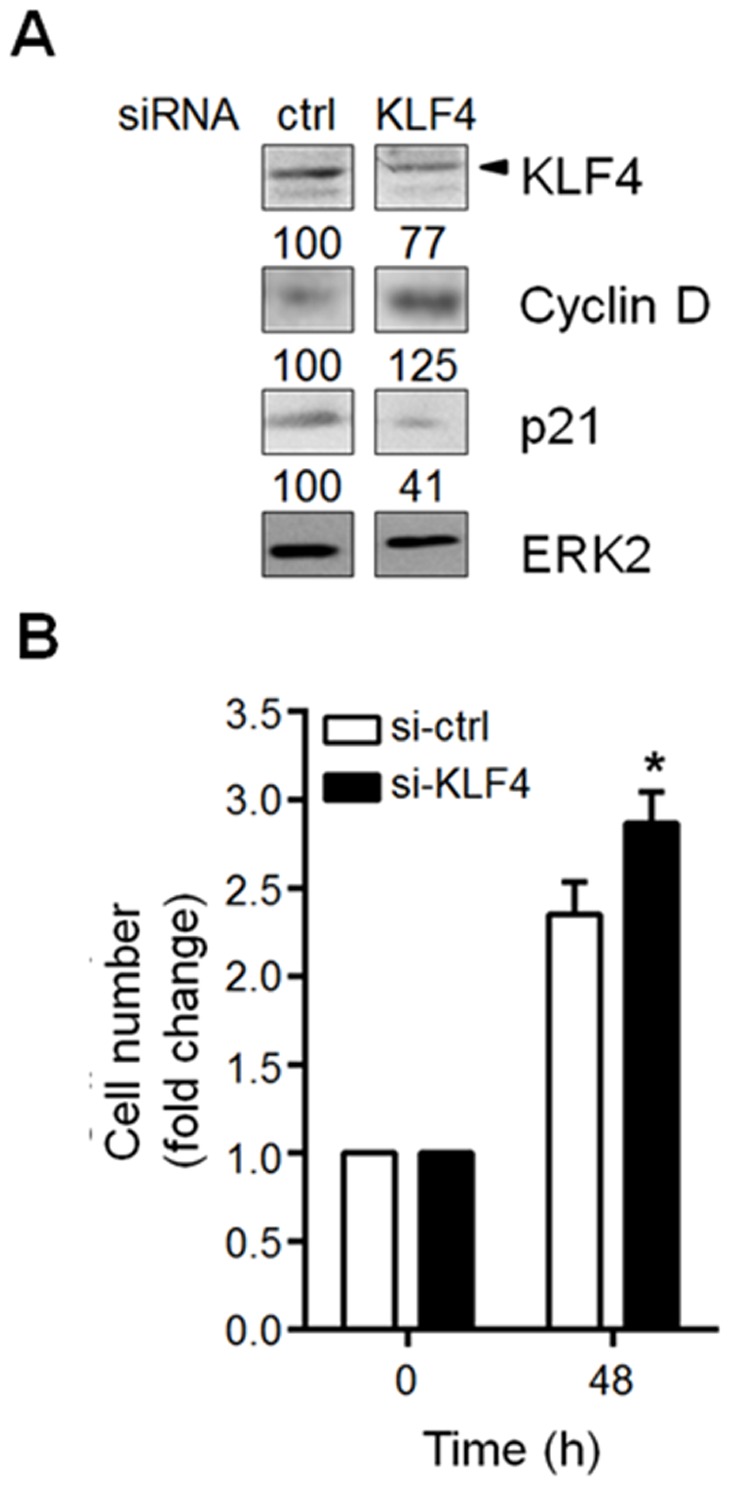
KLF4 downregulation in A549 cells enhances cell proliferation. (A) A549 cells were transfected with the KLF4 specific siRNAs (si-KLF4) or unspecific siRNAs (si-ctrl). 48 hours post-transfection total cell extracts were prepared and KLF4, Cyclin D and p21 protein levels were evaluated by Western blot. ERK2 protein was used as loading control. Numbers denote the protein levels as the percentage in si-KLF4 transfected cells relative to si-ctrl transfected cells. (B) The proliferative potential of A549 cells transfected with either si-KLF4 or si-ctrl was evaluated by counting cell number at the indicated time points. Data represent the mean of three independent experiments. *p<0.05 *vs.* si-ctrl at 48 hours.

KLF4 regulates cell cycle regulators such as *cyclin D* [14], *p21* [15], [16] and *p27* [15]. Therefore, we asked whether the increased proliferative capacity of cells overexpressing miR-7 could result from altered expression of KLF4 targets involved in cell cycle control. According with this idea, miR-7 expression prevented the time-dependent decline of Cyclin D protein levels and delayed the increase of p27 protein levels observed in confluent pcDNA transfected cells (Figure 3C and Figure S6). To further demonstrate that KLF4 downregulation results in increased cell proliferation, we reduced KLF4 protein levels by siRNAs in A549 cells. Transfection of the specific siRNAs for KLF4 resulted in a clear reduction of KLF4 protein levels 48 hours after transfection compared with cells transfected with nonspecific siRNAs (Figure 4A). Accordingly, Cyclin D protein levels increased while p21 protein levels were reduced compared with those observed in cells expressing normal KLF4 protein levels (ctrl) (Figure 4A). In agreement with the increase in Cyclin D and the reduction in p21 protein levels, cells transfected with the KLF4 specific siRNAs showed an enhanced proliferation capacity compared with control siRNAs transfected cells (Figure 4B). Together, our data indicate that miR-7, through reducing KLF4 protein levels, alters the protein levels of key regulators of the cell cycle resulting in enhanced cell proliferation of epithelial cells under space limiting conditions.

### miR-7 promotes migration of HaCaT and A549 cells

**Figure 5:**
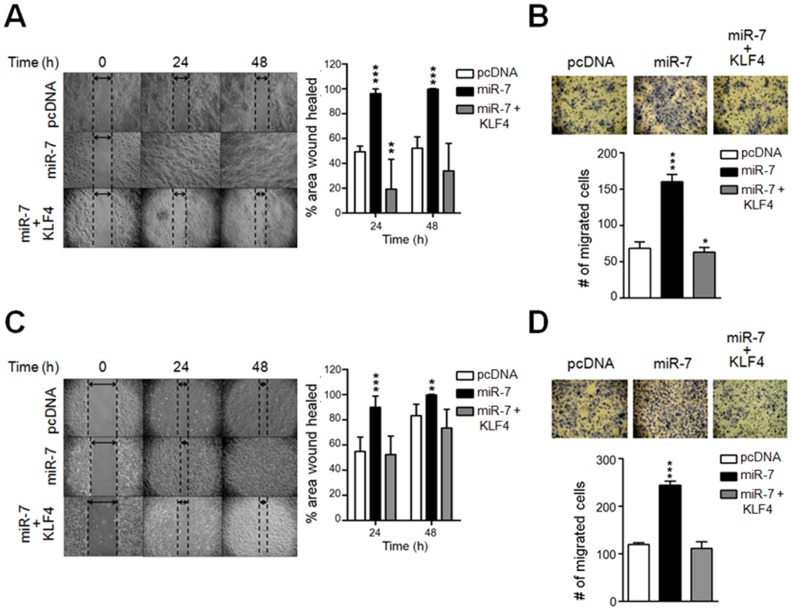
miR-7 overexpression induces wound-healing and migration capacities of HaCaT and A549 cells. Wound-healing assays were performed as described in materials and methods. HaCaT (A, left panel) or A549 cells (C, left panel) transfected with the pcDNA vector, expressing miR-7 only or both miR-7 and KLF4 (miR-7+KLF4) were photographed at the indicated times. The percentage of the wound-healed area at these time points was quantified using the TScratch software and plotted as the average of all the analyzed clones (A, C right panel). Migration assays were performed as described in materials and methods. Sixteen hours after plating, cells were photographed (B, D upper panel). The number of HaCaT (B lower panel) or A549 cells (D lower panel) transfected with the pcDNA vector, expressing miR-7 only or both miR-7 and KLF4 (miR-7+KLF4) was quantified and plotted. Data represents the average of three independent experiments performed with at least three independent clones of each analyzed condition. ***p<0.001, **p<0.01, *p<0.05 *vs.* pcDNA.

Given that miR-7 promotes cell proliferation and survival, we evaluated cell migration as another hallmark of cell transformation [49]. HaCaT or A549 cells expressing miR-7 were subjected to wound-healing assays to determine their migration potential. Interestingly, both HaCaT and A549 miR-7 expressing cells completely closed the wounded area around 24 hours later, while after 48 hours, pcDNA transfected cells only healed around 50% of the wounded area (Figure 5A and 5C and Movies S1, S2, S4 and S5). As expected, KLF4 expression prevented the miR-7 induced wound-healing capacity in both HaCaT and A549 cells (Figure 5A and 5C and Movie S3). Furthermore, KLF4 reduced the healing capacity of HaCaT cells below normal levels, since KLF4 expressing clones healed half of the area compared to that healed by the pcDNA transfected clones (Figure 5A, right panel). As wound healing might result from an increased proliferative capacity and/or higher cell motility, we performed migration assays. Consistently, miR-7 expressing cells showed an enhanced (2.5-fold) migratory capacity when compared to pcDNA transfected cells, independently of the cell type (Figure 5B and 5D). According to the data presented above, KLF4 co-expression reverted miR-7-induced motility in HaCaT and A549 cells (Figure 5B and 5D, lower panels). These results indicate that miR-7 positively regulates the motility and migration of epithelial cell lines and that this effect may be at least partly achieved by targeting KLF4.

### miR-7 promotes colony formation in vitro and tumor growth of A549 cells *in vivo*

**Figure 6:**
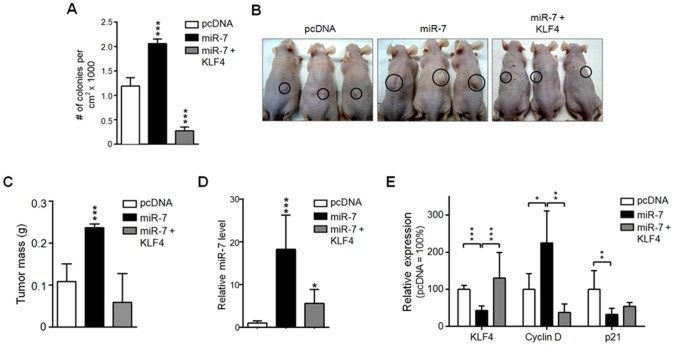
miR-7 overexpression promotes colony formation *in vitro* and tumor formation *in vivo* through KLF4 inhibition and regulating Cyclin D and p21 protein levels. (A) 1×10^5^ cells of three independent clones of A549 cells stably expressing pcDNA, miR-7 or miR-7+KLF4 were plated in a soft agar matrix and formed colonies were counted. (B) 3×10^6^ cells of three independent pcDNA, miR-7 or miR-7+KLF4 overexpressing A549 clones were subcutaneously injected into nude mice and after one-month post-implantation, mice were sacrificed. (C) Tumors were dissected and their mass was determined. (D) The relative expression of miR-7 on the tumors was determined by qPCR. (E) Tumors were analyzed for protein levels of KLF4, Cyclin D and p21 by Western blot assays using specific antibodies. ERK2 protein was used as loading control. The relative expression of each protein was calculated by dividing its densitometric signal by the ERK2 signal. Data were normalized considering the value of pcDNA transfected cells-derived tumors as 100%. Data represent the mean of three independent experiments using three (7 tumors), four (12 tumors) or three (8 tumors) independent miR-7, pcDNA or miR-7+KLF4 overexpressing clones, respectively. ***p<0.001, **p<0.01, *p<0.05 *vs.* pcDNA.

Given the increased proliferation and motility rates of HaCaT and A549 cells overexpressing miR-7, we further evaluated whether miR-7 could promote anchorage-independent growth, another hallmark of cell transformation. For that, the capacity of stably expressing pcDNA, miR-7 and miR-7+KLF4 A549 cells to form colonies in soft agar was tested. In agreement with the results presented above, miR-7 expressing cells formed more colonies when compared to pcDNA transfected cells (Figure 6A). Furthermore, expressing the miR-7 together with KLF4 reduced miR-7 effect on the number of colonies formed in soft agar even below the number of colonies observed in pcDNA transfected cells (Figure 6A). Thus, miR-7 promotes cell anchorage-independent growth *in vitro* suggesting an important role of miR-7 in epithelial cell transformation. To confirm this, we assessed miR-7 potential to promote tumor growth in an *in vivo* model. Different pcDNA, miR-7 and miR-7+KLF4 expressing A549 clones were subcutaneously injected into nude mice. All mice developed tumors independently of the clone; however, miR-7 expressing A549 cells began to form tumors 7 days post-implantation, while tumors derived from pcDNA cells were apparent only two weeks after injection. Consistent with this, tumors derived from miR-7 expressing cells at 30 days after seeding were bigger than those from pcDNA expressing cells (Figure 6B). The tumors derived from miR-7 expressing clones showed a significant increase in their mass compared to the tumors derived from pcDNA and miR-7+KLF4 transfected cells (Figure 6C). Likewise, tumors derived from miR-7 expressing cells contained lower KLF4 protein levels (Figure 6E and Figure S7). This was consistent with the fact that these tumors showed higher miR-7 levels than tumors derived from pcDNA transfected cells as determined by qPCR (Figure 6D). Accordingly with the reduced KLF4 protein levels, tumors derived from miR-7 expressing cells showed enhanced Cyclin D and diminished p21 protein levels (Figure 6E and Figure S7). Consistently, KLF4 expression reduced Cyclin D and increased p21 protein levels in tumors derived from miR-7+KLF4 expressing clones, compared with those observed in tumors derived from clones expressing miR-7 alone (Figure 6E and Figure S7). This effect was not due to the lack of miR-7 expression in miR-7+KLF4 expressing clones (Figure 6D). Together, these data indicate that miR-7 promotes tumor progression by targeting KLF4 and indicate that miR-7 acts as an oncomiR in lung epithelial cells.

## Discussion

miRNAs are key components of intricate gene expression regulatory networks involved in different biological processes including development and cell differentiation [29], [32], [50], [51], fat metabolism [52], immunity [53], cell cycle [54], cell death [55] and cancer [34], [56]. It is well known that in several types of cancer the expression pattern of specific miRNAs is altered [34]. Due to their regulatory role on different signal transduction pathways, miRNAs can have either a tumor suppressor or an oncogenic role during cancer development and progression [35], [57], [58]. Therefore, deregulation of these post-transcriptional regulators results in the altered expression of their direct target genes and consequently erroneous expression of downstream genes. Particularly, an altered expression profile of oncogenes, tumor suppressor genes and cell cycle regulatory genes results in a high risk of developing cancer.

KLF4 is a TF that can act as a tumor suppressor or as an oncogene [10]. Accordingly, low levels of KLF4 mRNA or protein have been particularly encountered in cancers of different epithelia [19]–[25]. In normal conditions, KLF4 represses the Wnt signaling by interacting with β-catenin in the nucleus [59]; preventing the transcription of genes such as *cyclin D* and *c-myc* which regulate the G1 to S phase transition of the cell cycle and therefore, cell proliferation [60]. However, in colorectal cancer the KLF4:β-catenin interaction is lost due to KLF4 downregulation causing de-repression of the Wnt signaling and uncontrolled cell proliferation [59]. Although hypermethylation and loss-of-heterozygosity have been reported as causative events for KLF4 downregulation in the intestinal epithelium, the molecular mechanisms responsible for KLF4 downregulation in cancer of other epithelial tissues have been poorly explored. In this sense miRNAs and especially oncomiRs, could exert specific downregulation of KLF4 in the epithelial context. Consistent with this idea, in this study, we show that miR-7 increases epithelial cell proliferation and migration rates by targeting KLF4 and consequently by altering the expression profile of cell cycle regulatory genes such as Cyclin D, p21 and p27. We also demonstrate that overexpression of miR-7 in epithelial cells promotes tumor formation in nude mice and that KLF4 protein levels are significantly downregulated in the formed tumors.

In addition to all miRNAs reported so far to target KLF4, our bioinformatics analyses predicted that miR-7 could also target KLF4 through two putative binding sites within the KLF4 3′ UTR. Our results from the luciferase reporter assays and western blot analyses demonstrated that miR-7 directly interacts with the KLF4 3′ UTR in a specific fashion mediating KLF4 protein level downregulation. Consistent with the fact that the second seed (574–580 nt) shows better thermodynamic stability to interact with the target mRNA and is conserved through evolution, mutation of this seed on the KLF4 3′ UTR abolished the decrease in luciferase activity resulted from miR-7 overexpression; even though, the first seed was intact. Hence, miR-7 negative effect on KLF4 protein levels is mediated through its interaction with an evolutionary conserved seed (seed 2) on the KLF4 3′ UTR. This seed presents a single mismatch while, the seed sequences recognized by other miRNAs that regulate KLF4 (miR-1 [42], miR-103/107 [37], miR-145 [43], miR-146 [41], miR-206 [36], miR-92a [61] and miR-10b [35]) present a mismatch and a wobble G:U pairing. Therefore, the specific and effective negative action of miR-7 over KLF4 expression is in accordance with the higher degree of sequence complementarity between miR-7 and its second binding site in the KLF4 3′ UTR compared to other KLF4 miRNA regulators. In addition, the functionality of this miR-7 seed sequence was also corroborated by other group in a breast cancer context [38].

According to the fact that KLF4 has a tumor suppressor function in epithelial cells [10], [62], here we show that down regulation of KLF4 protein by miR-7 overexpression in skin and lung epithelial cells promoted cell proliferation. The enhanced proliferative capacity of miR-7 expressing HaCaT cells resulted from increased entry into the cell cycle since higher number of cells in the S and in the G2/M phases were detected after growth factors addition. In contrast, miR-7 expressing A549 cells showed enhanced cell proliferation rate only when cultures reached confluence suggestive that miR-7 expression in A549 cells promotes both cell proliferation and cell survival. The difference in the effects of miR-7 expression on the proliferative capacity of these epithelial cell lines may result from the fact that A549 is a cell line derived from a human alveolar tumor [63] while HaCaT cells are naturally immortalized skin cells [64]; thus, A549 cells have a higher intrinsic proliferative capacity than HaCaT cells. Nonetheless, co-expression of KLF4 together with miR-7 reversed miR-7-dependent enhanced proliferation suggesting that this miRNA could act as an oncomiR in epithelial cell contexts by specifically targeting KLF4. The increased proliferative capacity of cells with low KLF4 protein levels as a consequence of miR-7 expression could result from altered expression of KLF4 targets involved in cell cycle control. It is known that KLF4 represses the transcription of cell cycle inductors such as *cyclin D* [13], [14], *cyclin B1* [65] and *cyclin E* [66] and it is also capable of inducing the transcription of the cell cycle inhibitors *p21* [15], [16], *p27* [15] and *p57* [67]. Under space limiting conditions, growth factors and nutrients begin to be scarce and starvation occurs resulting in a significant decrease in the cell proliferation rate characterized by a decrease in Cyclin D levels [68]. Under these conditions, miR-7 overexpression in A549 lung epithelial cells caused a delay in both, Cyclin D downregulation and induction of p27 protein levels. In contrast, levels of p21 appear not to be regulated by the miR-7:KLF4 axis under these experimental conditions as p21 protein levels are not downregulated when miR-7 is overexpressed (data not shown). The fact that down modulating KLF4 levels in A549 cells by specific siRNAs also resulted in enhanced cell proliferation further supports the idea that miR-7-induced cell proliferation and cell transformation involves KLF4 negative regulation (Figure 4). These data indicate that even at limiting growth factor conditions, miR-7 overexpression maintain cell proliferative state by preventing KLF4-mediated repression and induction of Cyclin D and p27 expression, respectively.

In addition to an enhanced cell proliferation rate, other hallmarks of the transformation process include higher motility and migration [49], [69]. Interestingly, miRNA deregulation has been reported to be one of the signals that can stimulate these processes [35]. In accordance with the increased proliferation rate observed in miR-7 overexpressing cells, both skin and lung epithelial cells overexpressing miR-7 showed increased motility as determined by the wound healing and migration assays. Strikingly, the migration capacity induced by miR-7 was prevented when KLF4 was co-expressed. However, the KLF4 downstream genes that inhibit the migratory capacity of epithelial cells are currently unidentified. So far, it is known that KLF4 acts as a tumor suppressor by positively regulating the levels of E-cadherin in hepatocellular carcinoma [62], MMP-2 and MMP-9 metalloproteases in neuroblastoma [70] and the TF Slug during the epithelial to mesenchymal transition of mammary epithelial cells [71]. In contrast, when KLF4 acts as an oncogene in breast CSCs, it promotes cell motility by activating the expression of some members of the Notch signaling pathway, particularly, Notch1, Notch2 and Jagged1 [72]. Therefore, whether these and/or other KLF4 target genes are involved in the migratory capacity of epithelial cells as result of miR-7 expression, must be determined.

According with the enhanced epithelial cell proliferation and migration resulted from miR-7-mediated KLF4 downregulation, miR-7 overexpression in A549 lung epithelial cells also promoted colony formation in soft agar and tumor formation in nude mice. The miR-7 induced tumors showed bigger size compared to those generated by the injection of empty vector transfected cells. Accordingly with the data presented above, tumors derived from miR-7 expressing cells showed decreased levels of KLF4 protein as well as altered protein levels of KLF4 target genes; Cyclin D and p21 protein levels were increased and decreased respectively which suggest that miR-7 acts as an oncomiR in epithelial cells. The fact that all these events were reversed when KLF4 was expressed together with miR-7 suggest that by directly targeting KLF4, miR-7 may contribute to an accelerated cell cycle progression and to an enhanced migratory capability of epithelial cells that result in a tumorigenic phenotype as a consequence of altered expression of KLF4 downstream targets involved in cell cycle and cell migration control.

In contrast to our results, another study using breast CSCs suggested that by negatively modulating KLF4 levels, miR-7 functions as a tumor suppressor. In this cellular context, KLF4 acts as an oncogene by promoting CSCs self-renewal and invasion abilities favoring the metastatic potential of these stem-like cells in an *in vivo* model [38]. This is not surprising as it is well known that KLF4 acts as an oncogene during breast cancer progression [26]. From these studies and the data reported here is evident that the miR-7 mediated cellular response depends on the cellular context. miR-7 overexpression in CHO cells triggers cell cycle arrest at the G1/S transition by targeting genes like Psme3 and Rad54L resulting in a deregulated expression of their downstream target genes including an increase in p27 and downregulation of Csk1, Cdk1/2 and Cyclin D1/3 [73]. miR-7 also promotes apoptosis of tumorigenic cells through regulating other targets than KLF4 including the anti-apoptotic protein BCL-2 [74]. Thus, whether miR-7 acts as a tumor suppressor or as an oncomiR will depend on the specific targeted genes, cellular context, growth conditions and the epigenetic background of individual cells. Nonetheless, our data showing that miR-7 expression promotes A549 cells tumorigenic capacity are in sharp contrast with a recent study showing that the negative regulation of BCL-2 by miR-7 inhibits proliferation, migration and tumorigenic capacities of miR-7 overexpressing A549 cells [74]. Although BCL-2 might be a *bona fide* miR-7 target, the fact that miR-7 overexpression only resulted in a 20% reduction of luciferase activity when using the BCL-2 3′ UTR compared to the 70% decrease in luciferase activity that we observed with the KLF4 3′ UTR suggests that the affinity of miR-7 for these two 3′ UTRs might be different. In addition, the fact that Xiong et al. used A549 transiently transfected with miR-7 and do not show miR-7 expression or BCL-2 protein levels in the tumors derived from the miR-7 expressing A549 cells, raises the possibility that miR-7 expression in those cells is not sustained for the duration of the assay and thus the observed effect may be independent of miR-7.

In conclusion, our findings that miR-7 negatively regulates the expression of the tumor suppressor KLF4 and that miR-7 overexpression promotes proliferation and migration of epithelial cells resulting in tumor formation *in vivo*, provide a mechanistic explanation for the aggressiveness of skin and lung tumors in which protein levels of KLF4 and Cyclin D have been shown to be down- and up-regulated, respectively [24], [25], [75]. Nonetheless, further experiments are required to show a negative correlation between miR-7 expression and KLF4 protein levels in samples of human epithelial tumors; this would be key to determine whether miR-7 could serve as a biomarker for the prognosis of epithelial cancer as miR-21 for gastric cancer patients [76].

## Materials and Methods

### Ethics Statement

*nu/nu* mice were maintained in our animal facility in a ventilated rack with food and water *ad libitum*. Experiments were carried according to institutional guidelines and to protocol N° 182 approved by the Bioethics Committee of the Instituto de Biotecnología, Universidad Nacional Autónoma de México.

### Bioinformatic prediction of target sites on the 3′ UTR of KLF4

All miRNAs reported for human and the genomic sequence of KLF4 3′ UTR were respectively obtained from the miRBase database release 15 (http://www.mirbase.org/) [77] and the Ensembl release 57 (http://www.ensembLorg/index.html) [78]. Bioinformatic analyses considering key features of a functional miRNA:target interaction were performed by using different bioinformatic tools including: TargetScanHuman release 5.1 (http://www.targetscan.org/vert_50/) [46], PITA (http://genie.weizmann.ac.il/pubs/mir07/mir07_prediction.html) [44], RNAHybrid (http://bibiserv.techfak.uni-bielefeld.de/rnahybrid/submission.html) [45], PicTar (http://pictar.mdc-berlin.de/) [79] and miRanda (http://www.microrna.org/microrna/getMirnaForMdo) [47]. Conservation of individual miRNAs and their target sites on KLF4 3′ UTR within different organisms was evaluated with TargetScanHuman release 5.1. miRNA:target thermodynamic stability was analyzed using PITA which calculates the difference (ΔΔG) between the Gibbs free energy released from the miRNA:target duplex formation (ΔG_duplex_) and the lost Gibbs free energy because of the conformational change to make accessible the target site for miRNA binding (ΔG_open_). ΔΔG values less than −10 indicate a high probability of a biologically functional interaction [44]. PicTar, miRanda and RNAHybrid were additionally used to confirm the presence of perfect or almost perfect sequence complementarity between the miRNA seed sequence and the 3′ UTR of the target gene (a maximum of one mismatch or G:U match is allowed in this region). Results were intersected and only miRNAs that satisfied all mentioned criteria were considered as good candidates.

### Cell culture

Human embryonic kidney 293 (HEK-293) cells were grown in Dulbecco's modified Eagle's medium (DMEM) (Gibco) supplemented with 10% fetal bovine serum (FBS), 2 mM L-glutamine (Sigma), 50 U/ml penicillin and 50 µg/ml streptomycin (Invitrogen).

Human no differentiated keratinocytes HaCaT cell line and its variant stable cells were cultured in Advanced Roswell Park Memorial Institute (RPMI) 1640 (Gibco) medium supplemented with 2 mM L-glutamine, 50 U/ml penicillin and 50 µg/ml streptomycin.

The human alveolar adenocarcinoma A549 cell line was cultured in DMEM-F12 (Gibco) medium with 5% FBS, 2 mM L-glutamine, 50 U/ml penicillin and 50 µg/ml streptomycin. All cell lines were cultured at 37°C and 5% CO_2_. All cell lines used in this study were obtained from ATCC.

### RNA extraction and RT-PCR

Total RNA was isolated from dissected tumors or cells using TRIzol reagent (Invitrogen) or following the Chomczynski's protocol [80], respectively. RNA concentration was determined using a Nanodrop dispositive (Thermo Scientific). For semiquantitative RT-PCR assays, miRNAs' reverse transcription (RT) reactions were done using stem-loop primers designed as previously reported [81]. RT reactions for the small nucleolar RNA (sncRNA) U6 were performed with reverse primer previously described [82]. The stem-loop RT for miR-7 and U6 was carried out with 100 ng of total RNA for each double reaction (miR-7:U6) using thermostable M-MLV Reverse Transcriptase (Invitrogen) according to the Varkonyi-Gasic's protocol [81]. RT negative controls without enzyme or RNA were equally treated. PCR reactions for miR-7 and the sncRNA U6 were performed according to Varkonyi-Gasic protocol [81] using 25 cycles for miR-7 and 30 cycles for U6. For quantitative PCR assays, miRNAs' RT reactions were performed using the NCode miRNA First-Strand cDNA Synthesis Kit (Invitrogen) following the manufacturer instructions. A specific forward primer was designated for miR-7. The U6 primers used in this study have been previously reported [82]. PCR assays were performed accordingly to the Maxima SYBR Green/ROX qPCR Master Mix (Thermo Scientific) kit instructions at 55°C. The primers used for semiquantitative and qPCR assays are listed in Table S2.

### Plasmid constructs

To amplify the 3′ UTR of the mouse *Klf4* gene (ENSMUST00000107619/NM_010637), the 3′ UTR was flanked with 200 bp at both ends using primers designed with the Primer BLAST program to generate a PCR product of 1264 bp. Then, a second pair of primers were used to amplify a fragment of 975 bp from the 1264 bp template of the KLF4 3′ UTR. The 975 bp fragment was flanked by XhoI and PmeI restriction sites at 5′ and 3′, respectively, and cloned into the psiCHECK-2 vector (psiCHECK-2) (Promega) downstream of the *Renilla* luciferase (r-luc) reporter gene, this construct was named psi/KLF4. Primers to amplify pre-miRNAs were designed using Primer3 [83] taking into account that for adequate miRNA overexpression it is necessary to clone the pre-miRNA flanked by a minimum of 40 bp at each side [50]. pre-miRNAs mmu-pre-miR-7a-1 (ENSMUSG00000065434), mmu-pre-miR-145 (ENSMUST00000083658) and mmu-pre-miR-881 (ENSMUST00000104844) were amplified including BamHI and EcoRI restriction sites and subsequently cloned into the pcDNA 3.1/myc-His A vector (pcDNA) (Invitrogen). Resulting plasmids were designated as pc/miR7, pc/miR145 and pc/miR881. Plasmid DNA was subsequently isolated from recombinant colonies and analyzed by enzymatic restriction and sequencing to ensure authenticity and orientation of the inserts. Additionally, a mutant version of the 3′ UTR of KLF4 (psi/KLF4-Mut) was generated in which two nucleotides of the seed sequence for the second miR-7 binding site GTCTTCC were substituted by GTAATCC (base substitution underlined). psi/KLF4-Mut was generated using the QuikChange II Site-Directed Mutagenesis Kit (Agilent Technologies) and a mutagenic oligonucleotide with the base substitution (listed in Table S2). All primers were analyzed for their optimal Tm and primer-dimer formation with Perl Primer software [84]; the primer sequences are listed in Table S2.

### Transfection and luciferase assays

2.5×10^5^ HEK-293 or A549 cells were seeded in 35 mm culture plates. At 80–95% confluence, cells were transfected with either 100 ng of empty psiCHECK-2 vector (CONTROL), 100 ng of the psi/KLF4 construct (SAMPLE) or 100 ng of the psi/KLF4-Mut construct (SAMPLE) together either 200 ng of empty pcDNA vector or pc/miR7 or pc/miR145 or pc/miR881 using Lipofectamine 2000 (Invitrogen). Following 24 hours of transfection, cells were fed with fresh growth medium and cultured for additional 24 hours. 48 hours post-transfection luciferase activity was determined using the Dual-Luciferase Reporter Assay System Kit (Promega), following the manufacturer instructions. Relative luciferase units (RLUs) were calculated as the ratio of (SAMPLEmiR-X*_Renilla_*_/firefly_/SAMPLEpcDNA*_Renilla_*_/firefly_)/(CONTROLmiR-X*_Renilla_*_/firefly_/CONTROLpcDNA*_Renilla_*_/firefly_) in which SAMPLE refers to KLF4 or KLF4-Mut and CONTROL refers to psiCHECK-2. After RLUs were calculated for each miRNA all were normalized taking empty vector (pcDNA) data as 1.

### RNA interference

To reduce KLF4 protein levels in A549 cells, 5×10^5^ cells were seeded in 24 well plates and when reached 90% confluence they were transfected with either the ON-TARGETplus Human KLF4 (9314) siRNA – SMARTpool (Thermo Scientific) or the siGENOME Non-Targeting siRNA Pool #1 (Thermo Scientific) using Lipofectamine 2000. Cells were trypsinized, washed and counted 48 h post-transfection. Reduction of KLF4 protein levels was confirmed by immunoblot.

### Western blot analysis

KLF4 protein levels were evaluated by Western blot assays as previously described [85]. Briefly, cells were lysed in 100 µl cold triton lysis buffer (20 mM Tris pH 7.4, 137 mM NaCl, 25 mM β-glycerophosphate pH 7.4, 2 mM PPiNa, 2 mM EDTA pH 7.4, 1% Triton X-100, 10% glycerol) supplemented with 1 mM Na_3_VO_4_, 1 mM PMSF, 0.5 mM DTT and 1× complete protease inhibitor cocktail (Roche), for 15 min at 4°C. Lysates were spun at 14,000 r.p.m. for 10 min at 4°C and kept at −70°C until use. Protein concentration was determined using the Bradford reagent (Bio-Rad). Total cell extracts (40 µg) were resolved by SDS-PAGE and transferred onto nitrocellulose membranes (GE Healthcare). Membranes were blocked with 5% non-fat milk in Tris-buffer saline (TBS: 10 mM Tris pH 7.5, 150 mM NaCl) with 0.1% Tween-20 (Sigma) (TBS-T), followed by incubation with the indicated antibody diluted in TBS-T. After three washes with TBS-T, membranes were incubated with the appropriate secondary antibody coupled to HRP (Santa Cruz Biotechnology). Proteins were visualized by chemiluminescence (Perkin Elmer) following the manufacturer's instructions. All primary antibodies used in this study were from Santa Cruz Biotechnology, Inc: KLF4 (GKLF T-16, 1∶200), ERK2 (ERK2 C-14, 1∶4000), p21 (p21 C-19, 1∶500), p27 (p27 F-8, 1∶1000) and Cyclin D (Cyclin D M-20, 1∶1000).

### Generation of stable cell lines

1.6×10^5^ HaCaT or A549 cells were transfected with 3 µg of linearized pcDNA vector or linearized pc/miR7 using Lipofectamine 2000. After 4 hours, transfection medium was replaced with the corresponding fresh medium and incubated for further 24 hours. 48 hours post-transfection cells were trypsinized and plated in 100 mm culture dishes. Clones were obtained by Geneticin/G418 (Gibco) selection using 1 µg/mL for HaCaT and 800 ng/mL for A549 cells. Clones showing miR-7 overexpression and decreased KLF4 protein levels as determined by RT-PCR and Western blot respectively (Figure S3 and S4) were selected for further experiments. At least, three independent clones showing normal KLF4 (pcDNA) or reduced KLF4 protein levels (miR-7) from each cell line were used for all biological assays. Moreover, independent clones with high levels of both miR-7 and KLF4 (Figure S3 and S4) were selected for the rescue experiments (miR-7+KLF4).

### Cell proliferation

5×10^4^ HaCaT or 2×10^4^ A549 cells were seeded in 24 well plates and cultured for the indicated times in supplemented medium then, cells were trypsinized, washed and counted at the indicated time points.

For post-confluence cell proliferation assays, 5×10^4^ A549 cells were seeded in 24 well plates and allowed to reach a 100% confluent state (T = 0). 24 h after, cells reached confluence, fresh medium was added and cells were cultured for the indicated time periods, trypsinized, washed and counted.

### Bromodeoxyuridine incorporation assays

1×10^5^ HaCaT cells of the different stable clones were plated in Nunc Lab-Tek chambered coverglass (Thermo Scientific) in Advanced RPMI 1640 medium. Once the cells were attached, Advanced RPMI was substituted by non-supplemented normal RPMI medium and were cultured for 24 hours to induce cell cycle arrest, then cells were fed with Advanced RPMI 1640 medium. Twelve hours later, 10 µM of 5-Bromo-2′-deoxyuridine (BrdU) (Sigma) was added to each well and cells were further cultured for 12 hours. Cells were then rinsed twice with PBS, fixed with 2% paraformaldehyde (PFA) during 45 min and then rinsed again twice with PBS. Next, cell permeabilization was performed with 0.1% Triton X-100 (Sigma) in PBS for 30 min at room temperature. Cells were rinsed with PBS and then blocked with 10% bovine serum albumin (BSA) (Santa Cruz Biotechnology) in PBS for 1 hour at room temperature. After rinsing twice with PBS, cells were treated with 50 U/mL of DNAse for 15 min at 37°C and then, washed with PBS twice. Finally, cells were incubated with the primary antibody anti-BrdU (BrdU Bu20A, 1∶200 Santa Cruz Biotechnology, Inc.) in dilution buffer (5% BSA, 0.1% Triton X-100 in PBS) overnight at 4°C. Cells were washed with PBS three times and incubated with the secondary antibody (Alexa488, 1∶200, Invitrogen) in dilution buffer for 1 hour at room temperature. The immunofluorescent signal was examined using a Zeiss axiovert microscope. Three fields of each sample were randomly selected and photographed. The percentage of proliferating HaCaT (BrdU^+^) was determined by counting the green cells and dividing this number by the total number of cells in each field.

### Cell cycle analysis

1.6×10^5^ HaCaT stable cells were seeded in 35 mm cell culture dishes in Advanced RPMI 1640 medium. Once the cells were attached, Advanced RPMI was substituted by non-supplemented normal RPMI medium and were cultured for 24 hours to induce cell cycle arrest, cells were then fed with Advanced RPMI 1640 medium. Cells were harvested at 0, 6, 12 and 24 hours after arrest and stained with propidium iodide (PI) to determine their cell cycle profile by flow cytometry. Briefly, cells were trypsinized at the indicated times, centrifugated at 1200 r.p.m. for 5 min, resuspended in a low salt solution [3.75 mM polyethylene glycol (Sigma), 50 µg/mL PI (Sigma), 360 Kunitz units of RNAse A (Sigma), 0.1% Triton X-100 and 3.75 mM sodium citrate buffer pH 7.2] and incubated for 30 min at 4°C. Thereafter, a high salt solution (3.75 mM polyethylene glycol, 0.05 mg/mL PI, 0.1% Triton X-100 and 376 mM sodium chloride) was added and samples were maintained at 4°C until DNA content was determined by flow cytometry using the FACSCanto II (Becton Dickinson). Data were analyzed using the FlowJo software.

### Wound healing assays

4.5×10^5^ HaCaT or A549 stable cells were seeded in 35 mm cell culture dishes. At 100% confluence, cell layers were scratched using a plastic pipette tip. Wound healing of each stable clone was subsequently monitored and photographed at 0, 12, 24, 36 and 48 hours using a Nikon Eclipse inverted microscope. The percentage of the wound-healed area was determined using the TScratch software. Moreover, the wound healing process of pcDNA (Movie S1), miR-7 (Movie S2) and miR-7+KLF4 (Movie S3) HaCaT clones as well as that of the pcDNA (Movie S4) and miR-7 (Movie S5) A549 clones was recorded by using a Nikon TE300 inverted bioluminescence microscope (See Movies S1, S2, S3, S4, S5).

### Migration assays

5×10^4^ HaCaT or A549 stable cells were seeded into Millicell Hanging Cell Culture Inserts (Millipore, pore size 8.0 µm) non-supplemented normal RPMI medium or DMEM-F12 medium supplemented with 0.5% FBS, respectively. In the lower chamber the bottom side of the inserts was immersed in Advanced RPMI 1640 or DMEM-F12 medium with 20% FBS. Cells were allowed to attach and to migrate for 16 hours at 37°C. After that, the inserts were removed and the cells in both sides of them were washed with PBS twice. Thereafter, cells were fixed with 3.7% PFA for 2 min at room temperature, washed with PBS and permeabilized with 100% methanol for 20 min at room temperature. After two washes with PBS, cells were stained with 4% trypan blue (Gibco) for 15 min at room temperature and washed once with PBS. Then, the cells from the upper face of the filter were scraped off with cotton swabs. Inserts were additionally stained with 4% trypan blue for 5 min. Finally, inserts were washed with PBS twice, visualized and counted under a Nikon Eclipse inverted microscope.

### Soft agar colony formation assays

Colony formation in soft agar was performed as previously described [86]. Briefly, 1×10^5^ A549 cells from distinct clones transfected with pcDNA vector, miR-7 expression vector or miR-7+KLF4 vectors were plated in triplicate, grown in a soft-agar matrix and incubated for 28 days. Formed colonies for each of the analyzed conditions were counted under a light microscope.

### *In vivo* tumor formation

Six weeks old male *nu/nu* mice were inoculated subcutaneously with 3×10^6^ cells from different A549 clones containing the empty pcDNA vector, miR-7 or miR-7+KLF4 expression vectors. After one month, animals were sacrificed, each tumor was surgically excised and the mass determined. The levels of miR-7 as well as KLF4 and cell cycle regulators were determined by RT-qPCR and Western blot assays, respectively.

### Statistical analyses

Data are presented as mean ± standard deviation. Kolmogorov-Smirnov normality tests were applied to the data. For multiple paired comparisons Student's t tests were used to determine *p-values*. OpenOffice and Prism soft wares were used to perform all the statistical tests whose significance value was defined as ***p<0.001, **p<0.01, *p<0.05.

## Supporting Information

Figure S1 **miR-7 endogenous levels and effectiveness of miR-7 overexpression.** Measurement of endogenous miR-7 levels by qRT-PCR of HEK-293 and A549 cells (A). Measurement of miR-7 expression levels of HEK-293 and A549 transfected with either the empty vector (pcDNA) or the pc/miR7 construct (B). U6 was used as internal control for qRT-PCR. n = 3, ***p<0.01 *vs.* pcDNA; *p<0.05 *vs*. HEK-293.
(TIFF)

Figure S2 **miR-7 decreases endogenous KLF4 protein levels.** Whole cell lysates from HEK-293 cells transfected with the indicated amount of pc/miR7, pc/miR145 or pc/miR881 were analyzed to determine KLF4 protein levels by Western blot assays using a KLF4 specific antibody. ERK2 protein was used as loading control. The relative expression of KLF4 was calculated by dividing its densitometric signal by the ERK2 signal. All values were normalized considering the value of pcDNA (-) transfected cells as 1. n = 4, **p<0.01, *p<0.05 *vs.* pcDNA.
(TIFF)

Figure S3 **Generation of clones of HaCaT cells that stably overexpress miR-7 or miR-7+KLF4.** Measurement of miR-7 expression levels by stem-loop RT-PCR of stable clones of HaCaT cells that overexpress either empty vector (pcDNA), miR-7 or miR-7+KLF4 (A). Western blot to determine KLF4 protein levels in stable clones of HaCaT cells that overexpress pcDNA, miR-7 or miR-7+KLF4 (B). U6 and ERK2 were used as loading controls for RT-PCR and Western blot assays, respectively.
(TIFF)

Figure S4 **Generation of clones of A549 cells that stably overexpress miR-7 or miR-7+KLF4.** Measurement of miR-7 expression levels by stem-loop RT-PCR of stable clones of A549 cells that overexpress either empty vector (pcDNA), miR-7 or miR-7+KLF4 (A). Western blot to determine KLF4 protein levels in stable clones of A549 cells that overexpress pcDNA, miR-7 or miR-7+KLF4 (B). U6 and ERK2 were used as loading controls for RT-PCR and Western blot assays, respectively.
(TIFF)

Figure S5 **miR-7 overexpression promotes cell cycle progression of HaCaT cells.** 1.6×10^5^ HaCaT cells were seeded in 35 mm cell culture. Once attached, cells were deprived of growth factors for 24 hours to induce cell cycle arrest and then, growth factors were added. Cells were harvested at 0, 12 and 24 hours after arrest and stained with propidium iodide (PI) to determine their cell cycle profile by flow cytometry. h, hours.
(TIFF)

Figure S6 **miR-7 regulates the protein levels of KLF4 target genes.** Whole cell lysates from A549 cells stably overexpressing miR-7 or the empty vector (pcDNA) were analyzed to determine Cyclin D (CycD) (A) and p27 (B) protein levels by Western blot assays using specific antibodies. ERK2 protein was used as loading control. The relative expression of each protein was calculated by dividing its densitometric signal by the ERK2 signal. All values were normalized considering the value of pcDNA transfected cells at the 0 hours time point as 100%. Data represent the mean of three independent experiments, *p<0.05 *vs.* pcDNA.
(TIFF)

Figure S7 **miR-7 overexpression in tumors inhibits KLF4 and regulate Cyclin D and p21 protein levels.** Representative Western blots showing protein levels of KLF4, Cyclin D and p21 in a tumor derived from pcDNA transfected cells and three independent tumors derived from miR-7 overexpressing cells (A) and in pools of three independent tumors derived from either miR-7 or miR-7+KLF4 expressing clones (B). ERK2 protein was used as loading control.
(TIFF)

Table S1 **miRNAs with predicted binding sites within the KLF4 3′ UTR are listed with their ΔΔG values as calculated by PITA.**
(DOC)

Table S2 **RT-PCR and qPCR primers.**
(DOC)

Movie S1 **Wound healing process of a pcDNA HaCaT clone recorded by using a Nikon TE300 inverted bioluminescence microscope.**
(MPG)

Movie S2 **Wound healing process of a miR-7 HaCaT clone recorded by using a Nikon TE300 inverted bioluminescence microscope.**
(MPG)

Movie S3 **Wound healing process of a miR-7+KLF4 HaCaT clone recorded by using a Nikon TE300 inverted bioluminescence microscope.**
(MPG)

Movie S4 **Wound healing process of a pcDNA A549 clone recorded by using a Nikon TE300 inverted bioluminescence microscope.**
(MPG)

Movie S5 **Wound healing process of a miR-7 A549 clone recorded by using a Nikon TE300 inverted bioluminescence microscope.**
(MPG)
