# Supplementary figures and images for: MiR-7 Promotes Epithelial Cell Transformation by Targeting the Tumor Suppressor KLF4
Source: PLoS One. 2014 Sep 2;9(9):e103987. doi: 10.1371/journal.pone.0103987 (PMC4151986; doi:10.1371/journal.pone.0103987)

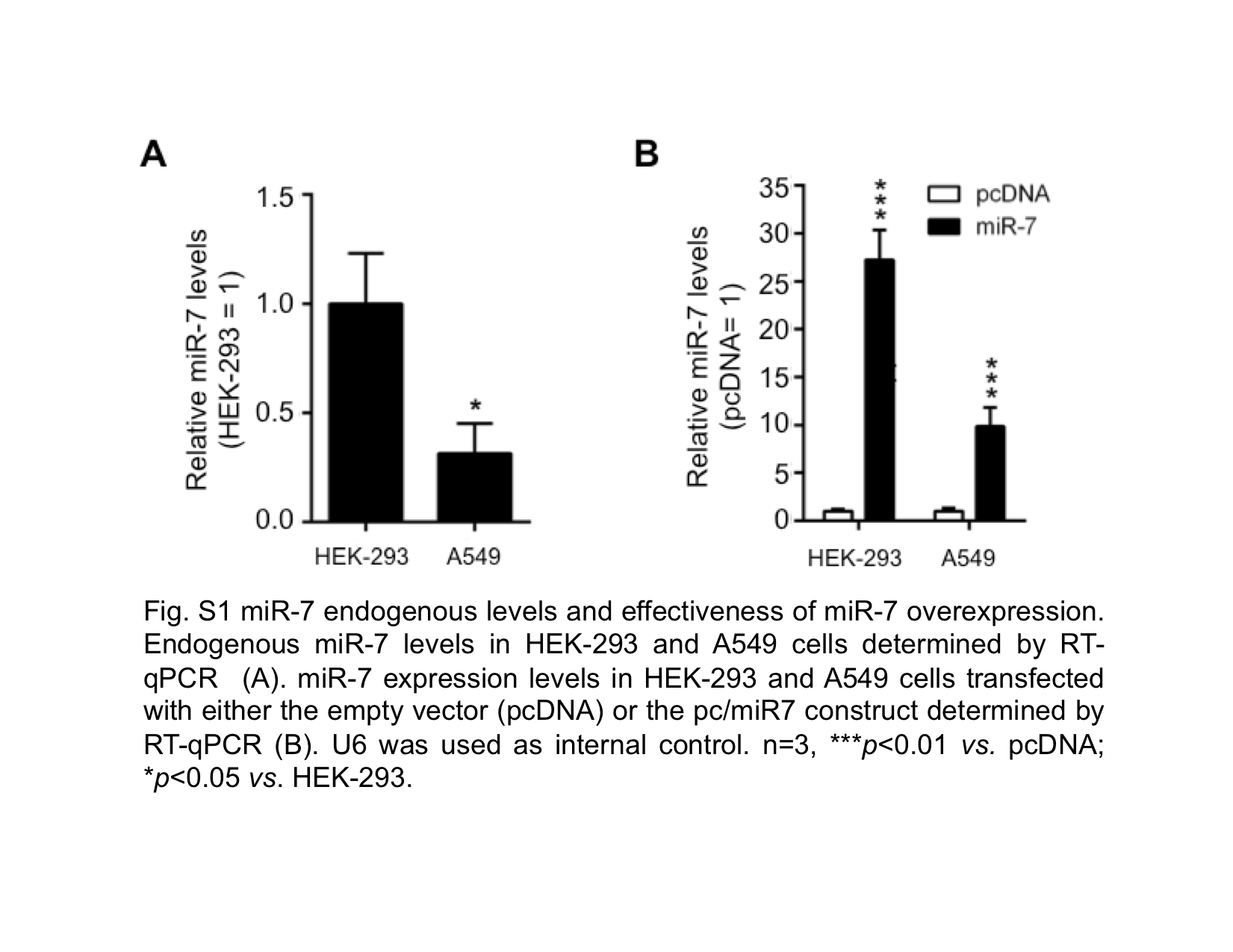

Supplement: Figure S1 — miR-7 endogenous levels and effectiveness of miR-7 overexpression. Measurement of endogenous miR-7 levels by qRT-PCR of HEK-293 and A549 cells (A). Measurement of miR-7 expression levels of HEK-293 and A549 transfected with either the empty vector (pcDNA) or the pc/miR7 construct (B). U6 was used as internal control for qRT-PCR. n = 3, ***p<0.01 vs. pcDNA; *p<0.05 vs. HEK-293. (TIFF) [file pone.0103987.s001.tiff]

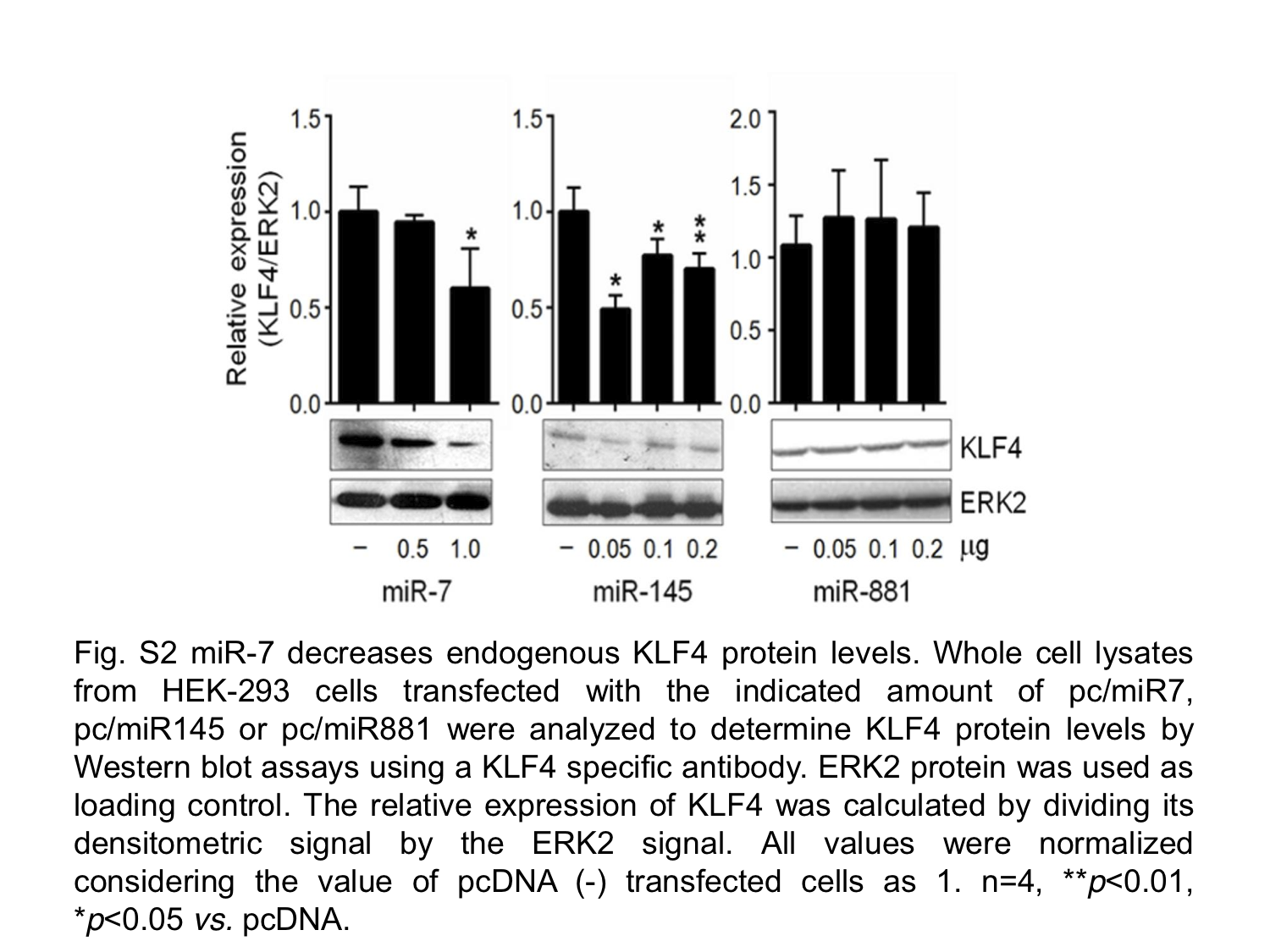

Supplement: Figure S2 — miR-7 decreases endogenous KLF4 protein levels. Whole cell lysates from HEK-293 cells transfected with the indicated amount of pc/miR7, pc/miR145 or pc/miR881 were analyzed to determine KLF4 protein levels by Western blot assays using a KLF4 specific antibody. ERK2 protein was used as loading control. The relative expression of KLF4 was calculated by dividing its densitometric signal by the ERK2 signal. All values were normalized considering the value of pcDNA (-) transfected cells as 1. n = 4, **p<0.01, *p<0.05 vs. pcDNA. (TIFF) [file pone.0103987.s002.tiff]

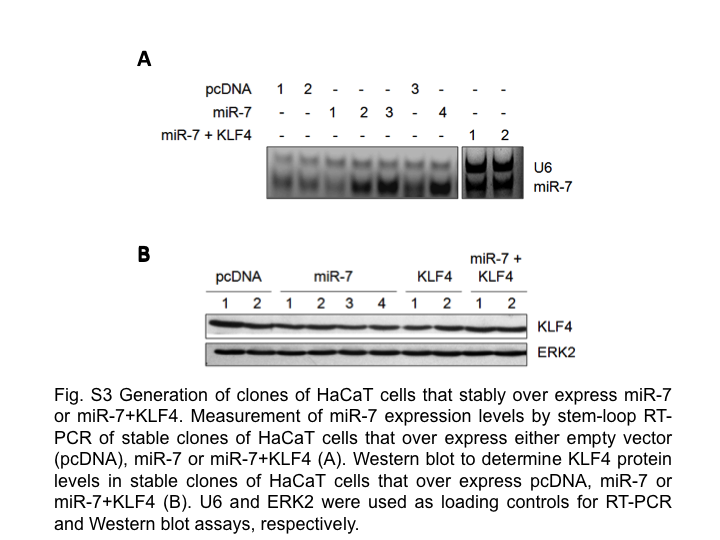

Supplement: Figure S3 — Generation of clones of HaCaT cells that stably overexpress miR-7 or miR-7+KLF4. Measurement of miR-7 expression levels by stem-loop RT-PCR of stable clones of HaCaT cells that overexpress either empty vector (pcDNA), miR-7 or miR-7+KLF4 (A). Western blot to determine KLF4 protein levels in stable clones of HaCaT cells that overexpress pcDNA, miR-7 or miR-7+KLF4 (B). U6 and ERK2 were used as loading controls for RT-PCR and Western blot assays, respectively. (TIFF) [file pone.0103987.s003.tiff]

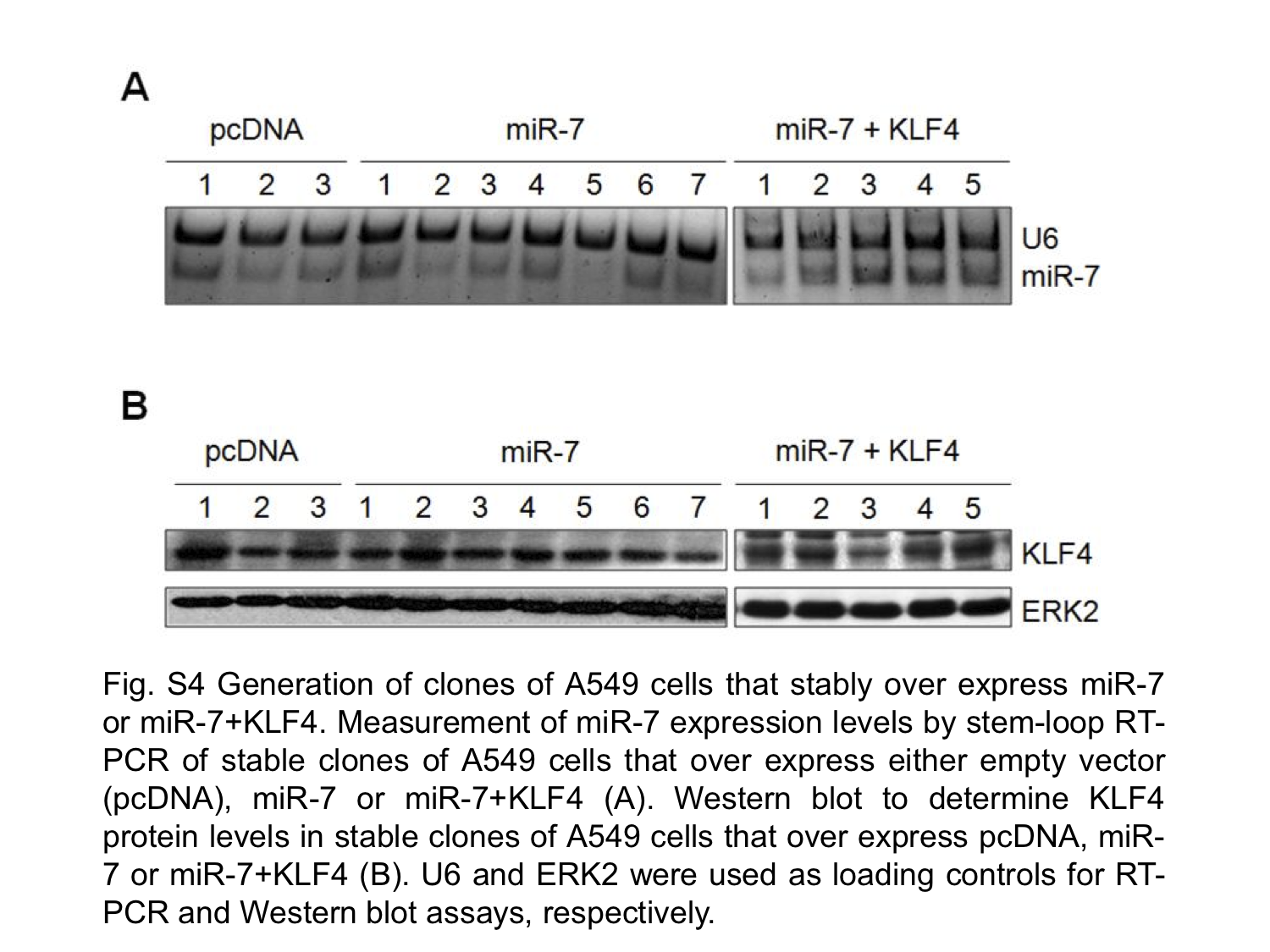

Supplement: Figure S4 — Generation of clones of A549 cells that stably overexpress miR-7 or miR-7+KLF4. Measurement of miR-7 expression levels by stem-loop RT-PCR of stable clones of A549 cells that overexpress either empty vector (pcDNA), miR-7 or miR-7+KLF4 (A). Western blot to determine KLF4 protein levels in stable clones of A549 cells that overexpress pcDNA, miR-7 or miR-7+KLF4 (B). U6 and ERK2 were used as loading controls for RT-PCR and Western blot assays, respectively. (TIFF) [file pone.0103987.s004.tiff]

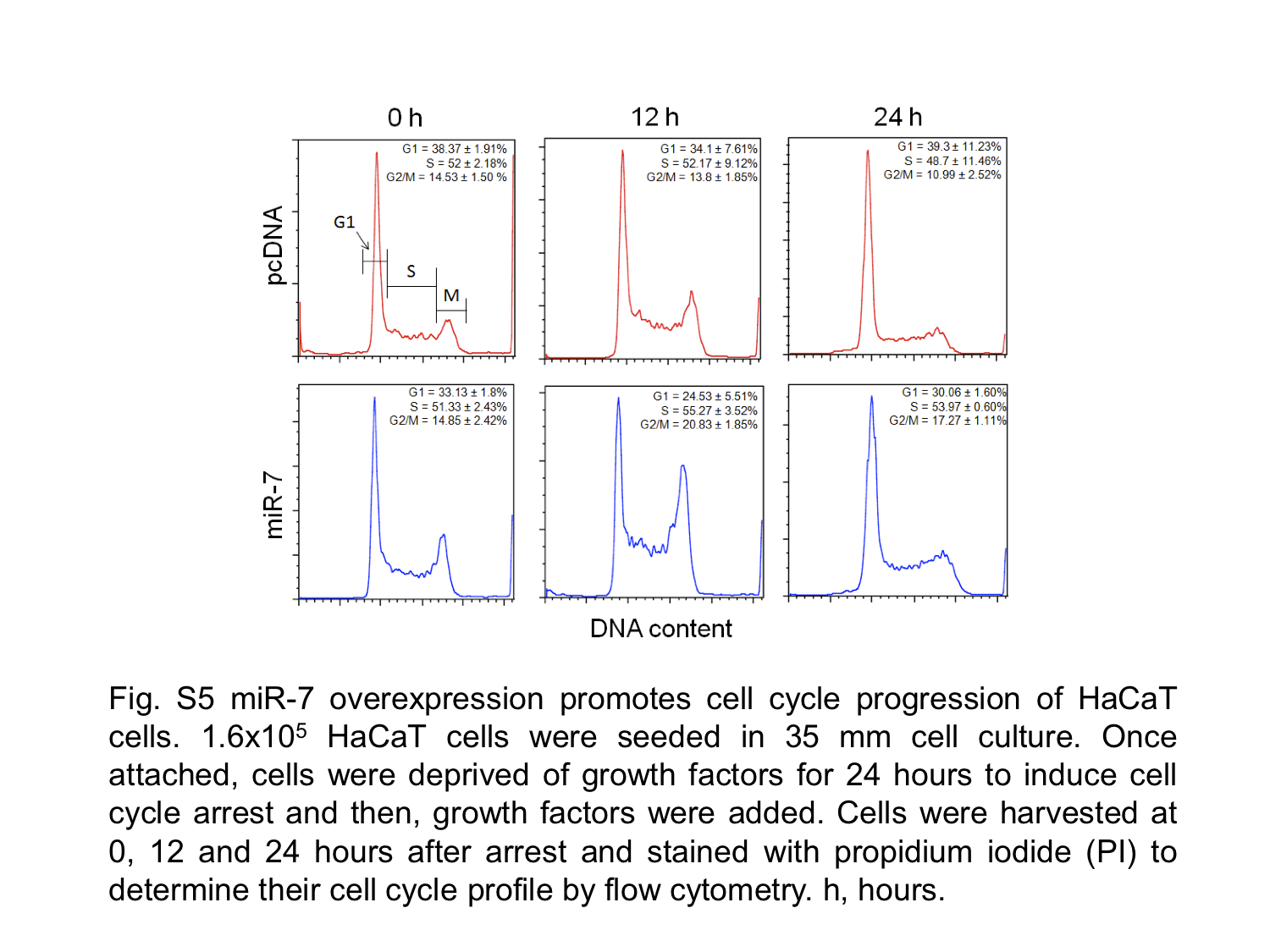

Supplement: Figure S5 — miR-7 overexpression promotes cell cycle progression of HaCaT cells. 1.6×10^5^ HaCaT cells were seeded in 35 mm cell culture. Once attached, cells were deprived of growth factors for 24 hours to induce cell cycle arrest and then, growth factors were added. Cells were harvested at 0, 12 and 24 hours after arrest and stained with propidium iodide (PI) to determine their cell cycle profile by flow cytometry. h, hours. (TIFF) [file pone.0103987.s005.tiff]

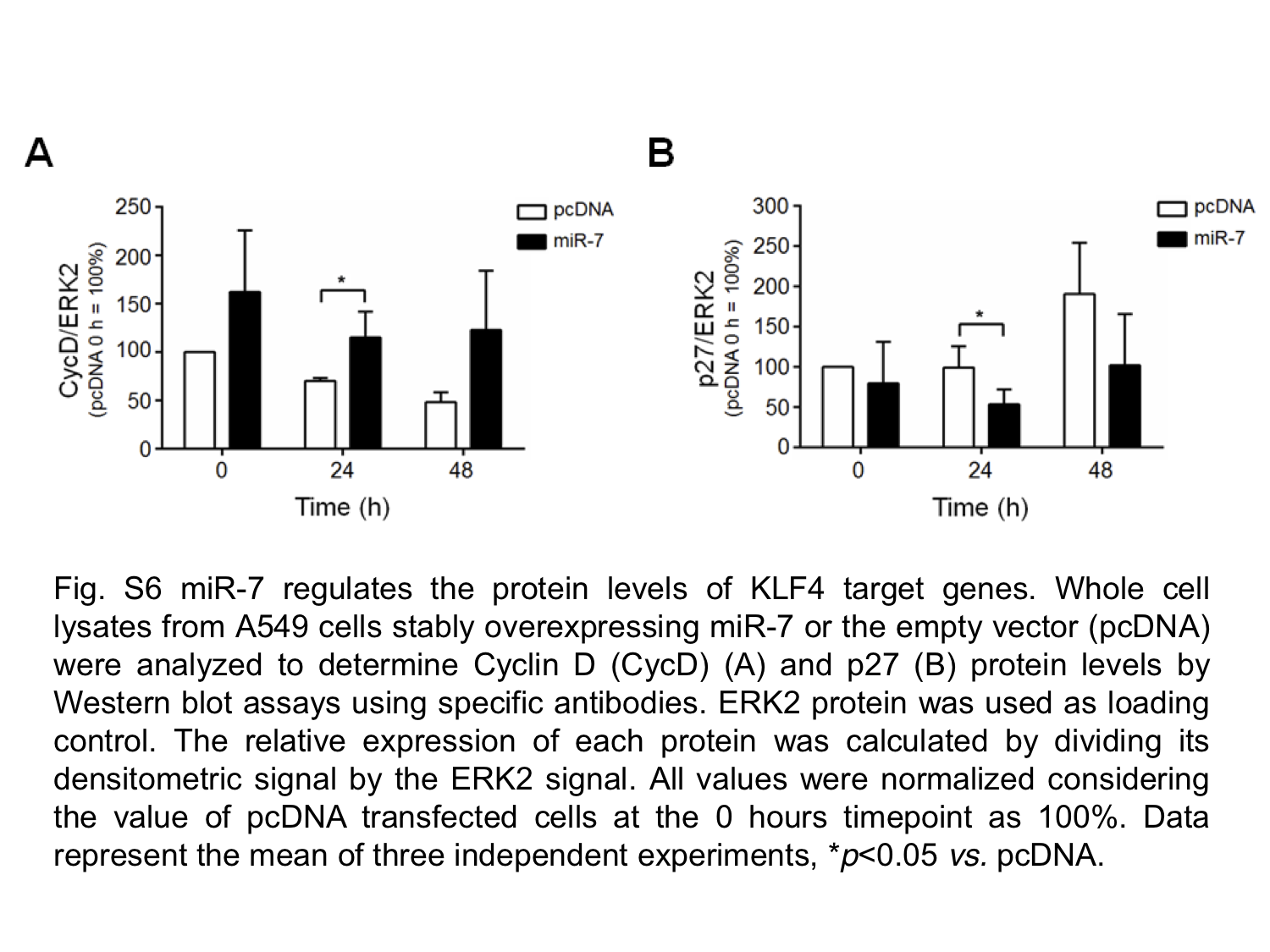

Supplement: Figure S6 — miR-7 regulates the protein levels of KLF4 target genes. Whole cell lysates from A549 cells stably overexpressing miR-7 or the empty vector (pcDNA) were analyzed to determine Cyclin D (CycD) (A) and p27 (B) protein levels by Western blot assays using specific antibodies. ERK2 protein was used as loading control. The relative expression of each protein was calculated by dividing its densitometric signal by the ERK2 signal. All values were normalized considering the value of pcDNA transfected cells at the 0 hours time point as 100%. Data represent the mean of three independent experiments, *p<0.05 vs. pcDNA. (TIFF) [file pone.0103987.s006.tiff]

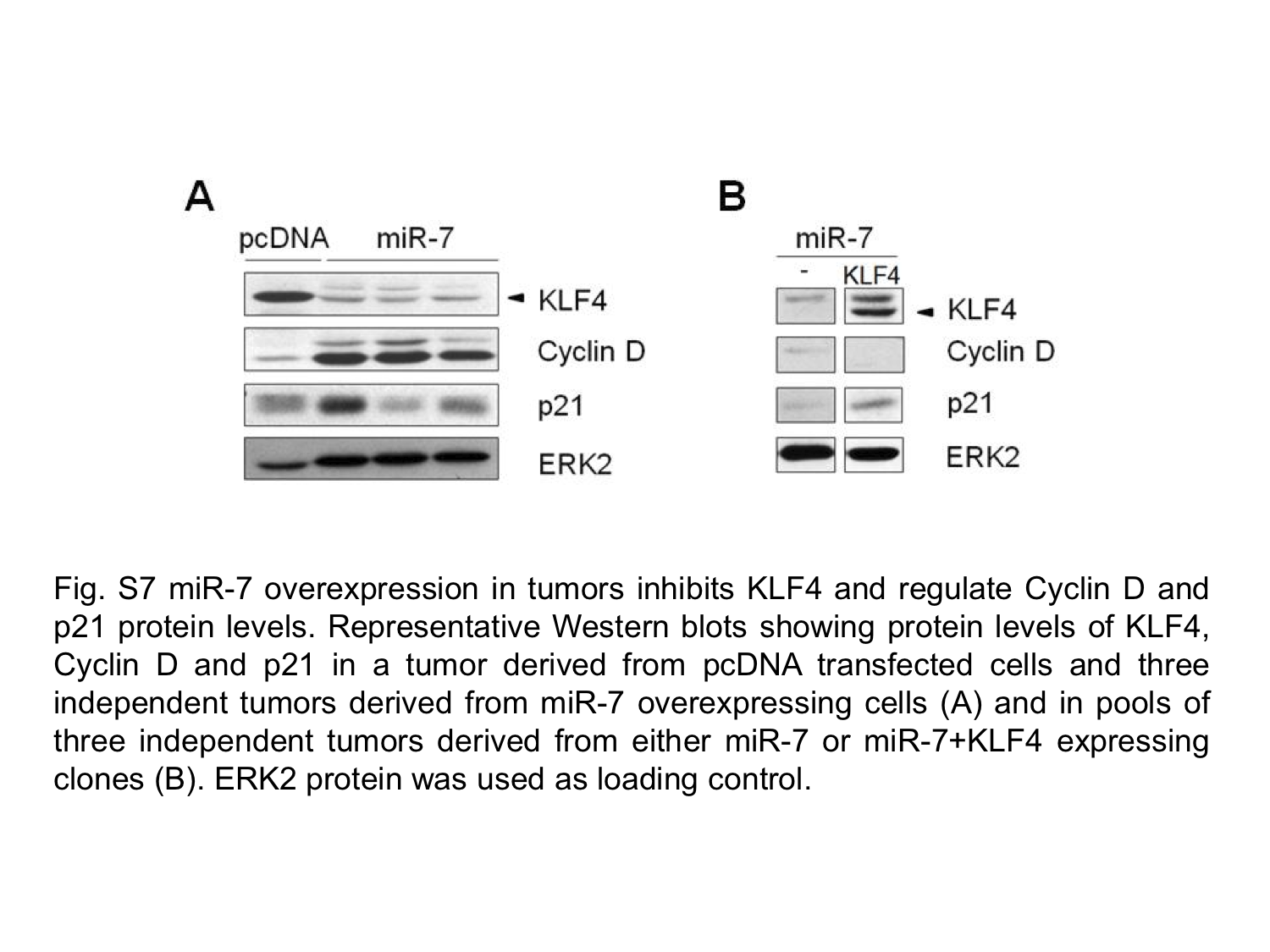

Supplement: Figure S7 — miR-7 overexpression in tumors inhibits KLF4 and regulate Cyclin D and p21 protein levels. Representative Western blots showing protein levels of KLF4, Cyclin D and p21 in a tumor derived from pcDNA transfected cells and three independent tumors derived from miR-7 overexpressing cells (A) and in pools of three independent tumors derived from either miR-7 or miR-7+KLF4 expressing clones (B). ERK2 protein was used as loading control. (TIFF) [file pone.0103987.s007.tiff]
