# Supplementary material for: MiR-7 Promotes Epithelial Cell Transformation by Targeting the Tumor Suppressor KLF4
Source: PLoS One. 2014 Sep 2;9(9):e103987. doi: 10.1371/journal.pone.0103987 (PMC4151986; doi:10.1371/journal.pone.0103987)
Supplement: Table S1 — miRNAs with predicted binding sites within the KLF4 3′ UTR are listed with their ΔΔG values as calculated by PITA. (DOC) [file pone.0103987.s008.doc]

Table S1. miRNAs with predicted binding sites within the KLF4 3’ UTR are listed with their ΔΔG values as calculated by PITA.

| miRNA | ΔΔG |
| --- | --- |
| miR-103/107/107ab | -7.73 |
| miR-148ab-3p/152 | -12.15 |
| miR-25/32/92abc/363/363-3p/367 | -9.42 |
| miR-29abcd | -8.44 |
| miR-7/7ab | -11.47 |
