# Supplementary material for: MiR-7 Promotes Epithelial Cell Transformation by Targeting the Tumor Suppressor KLF4
Source: PLoS One. 2014 Sep 2;9(9):e103987. doi: 10.1371/journal.pone.0103987 (PMC4151986; doi:10.1371/journal.pone.0103987)
Supplement: Table S2 — RT-PCR and qPCR primers. (DOC) [file pone.0103987.s009.doc]

Table S2. RT-PCR and qPCR primers.

| Gene | Primer Sequence (5' to 3') | | PCR product (nt) |
| --- | --- | --- | --- |
| mouse-KLF4 3’ UTR | Fwd : CAGGGCACCGGCCCTTTCAG  Rev: AGGTCCCATCCGTACAGCTCTCA | | 1264 |
| mouse-KLF4 3’ UTR | Fwd (XhoI): CTTGCTCGAGATCCCACGTAGTGGATGTG  Rev (PmeI): AGCTGCGTTTAAACCTTATTTCTCACCTTGAGTAT | | 975 |
| mouse-pre-miR-7a | Fwd (BamHI): TAGGATCCTGCTGCCAAAACCACCACTTGT  Rev (EcoRI): GCGAATTCTGCAGGGTTAAGAAAATGAAACTGGA | | 295 |
| mouse-pre-miR-145 | Fwd (BamHI): TAGGATCCGCTCTTCCCAGAGCAGGAC  Rev (EcoRI): GCGAATTCCAGTTCTGAGCTTCCCACATC | | 415 |
| mouse-pre-miR-881 | Fwd (BamHI): TAGGATCCGGACAGGCTGATGCTATTCC  Rev (EcoRI): GGGAATTCCCCCAATGACGATTGAAGTT | | 240 |
| human/mouse-sncRNA U6 | Fwd: CTCGCTTCGGCAGCACA  Rev: AACGCTTCACGAATTTGCGT | | 94 |
| miR-7 stem-loop RT primer | GTCGTATCCAGTGCAGGGTCCGAGGTATTCGCACTGGATACGACACAACA | | N/A |
| miR-7 | Fwd: GGGAGGTGGAAGACTAGTGATTT  Universal reverse primer: GTGCAGGGTCCGAGGT | | 63 |
| qPCR miR-7 | Fwd: TGGAAGACTAGTGATTTTGTTGT | | N/A |
| MutS2_KLF4 (2^nd^ seed for miR-7) | AATCTAGTTTTGTAATCCGATCTACATTG | | N/A |
| N/A, not applicable. | |  |  |
